# Impostor phenomenon short scale (IPSS-3): a novel measure to capture impostor feelings in large-scale and longitudinal surveys

**DOI:** 10.3389/fpsyg.2024.1358279

**Published:** 2024-11-12

**Authors:** Max P. Jansen

**Affiliations:** ^1^Faculty of Social Sciences, Goethe University Frankfurt, Frankfurt am Main, Germany; ^2^Institute for Social Research (IfS), Goethe University Frankfurt, Frankfurt am Main, Germany

**Keywords:** impostor phenomenon, short scale, large-scale surveys, longitudinal research, social identity, social context

## Abstract

The Imposter Phenomenon (IP) is gaining increasing attention in academia, not only as an overall attractive research topic but also as a concern that especially affects members of minority groups. Nevertheless, there is little evidence for the occurrence and socio-structural correlates of the IP. Against the backdrop of a pressing need to contextualize the IP, this paper provides (1) an overview of the existing empirical evidence on the IP from a perspective that incorporates the role of social contexts, (2) highlights shortcomings in both existing theoretical approaches and methodological tools, (3) introduces the Impostor Phenomenon Short Scale (IPSS-3) as a novel, time-efficient and universally applicable IP measure, and (4) underscores that the IP, in fact, does not occur in a social vacuum but is closely intertwined with socio-structural characteristics. To this end, the paper draws on three distinct data sets gathered among German adolescents and adults for the development of the IPSS-3 (Study 1: *n* = 271), its validation (Study 2: *n* = 427), and to assess the IP’s socio-structural correlates (Study 3: *n* = 865). The findings demonstrate that the IPSS-3 represents the first time-efficient and universally applicable instrument suitable for capturing the IP in large-scale and longitudinal research designs, e.g., initiated in adolescence. Thus, the IPSS-3 can address key open questions related to age effects, the role of transitions in the life course, and systematic variations in IP intensity among different social groups.

## 1 Introduction

Since first being described in 1978 by psychologists Clance and Imes (1978), a wealth of research has been published on the “Impostor Phenomenon” (IP) highlighting its devastating psychological effects, for example, in relation to anxiety, depression, and self-esteem (e.g., Sonnak and Towell, 2001; McGregor et al., 2008). This body of literature provides a comprehensive account of the detrimental consequences of the IP on an individual’s well-being and career advancement (e.g., Vergauwe et al., 2015; Neureiter and Traut-Mattausch, 2016; Noskeau et al., 2021).

While the IP generally refers to feelings of “inauthentic success” and “intellectual fraudulence” (Levesque, 2018), it is associated with the basic observation that individuals can feel intellectually inadequate despite objective proof of their competence and success (Clance and Imes, 1978). Experiencing high levels of the IP is associated with attributing achievements to external, unstable factors (e.g., good social contacts, luck, or coincidence) rather than internal stable abilities (Bradley, 1978; Ross et al., 2001; Bernard et al., 2018; Levesque, 2018). Moreover, an experimental study by Brauer and Proyer (2022) has demonstrated that the IP is related to the actual attributions of feedback after the successful completion of intelligence test tasks.

The IP was originally conceptualized in a multidimensional manner by Clance and Imes (1978) since it occurs at different levels, namely (1) constant doubts about one’s own abilities, (2) fears of being exposed (as an impostor), and (3) sentiments of social inadequacy expressed in feelings of not belonging/not conforming to other’s expectations, that act as barriers to social participation and advancement (Neureiter and Traut-Mattausch, 2016).

Against this background, self-help literature as well as increasing academic contributions have contributed to a flurry of interest that has made the IP an ever-popular topic (Cozzarelli and Major, 1990: 401; Feenstra et al., 2020; Anderson-Zorn, 2021).

As a result, the IP is subject to extensive debates in both academic and popular literature. However, most publications apply a merely psychological perspective on the IP and focus on the examination of personality-based factors, while differences based on socio-demographic characteristics are rarely taken into account. Studies on the IP often focus on specific study populations and often include no or small comparison groups, which is why cross-cultural equivalence so far has to be considered an absence of central importance in research on the IP (for a review see Bravata et al., 2019).

Moreover, the IP has so far been assessed almost exclusively via small-scale, cross-sectional surveys, usually based on highly selective (student) samples (for a review see Mak et al., 2019). This may explain why key questions regarding the overall occurrence and socio-structural correlates of the IP, thus, remain unanswered (Bravata et al., 2019; Feenstra et al., 2020).

Given the major socio-structural reconfigurations that have taken place since the IP was first described in 1978 (e.g., educational expansion, occupational upgrading, rising female employment and migration), it seems fruitful to foster a more nuanced understanding of the IP by taking into account the role of socio-structural contexts (Feenstra et al., 2020).

Against this background, the following section provides a brief overview of the current empirical evidence base on the IP to highlight key shortcomings from a perspective interested in the role of social contexts that are linked to the existing IP instruments. These instruments are described in more detail in the following section. Subsequently, the Impostor Phenomenon Short Scale (IPSS-3) as a time-efficient and universally applicable instrument suitable for studying the IP in large-scale and longitudinal surveys is introduced. Following this, the development and validation of the IPSS-3 is described in detail, based on two separate data sets (Study 1: *n* = 271; Study 2: *n* = 427), whereas a third data set is utilized to analyze the socio-structural correlates of the IP (Study 3: *n* = 865). Subsequently, the results are discussed highlighting limitations and potential directions for future research. The final section concludes with a concise summary of the findings, emphasizing the suitability of the IPSS-3 for future research endeavors aimed at understanding the role of societal, institutional, and interpersonal aspects in the experience of the IP.

## 2 A context-informed perspective on the IP

Currently, the IP is predominantly perceived as a feature of one’s personality (Feenstra et al., 2020). Against this backdrop, this chapter identifies aspects that largely stay out of focus to point out key areas worth examining to contextualize this trending phenomenon. Furthermore, major gaps in the existing IP research are outlined that should be worth addressing from a perspective interested in the role of social contexts in experiencing the IP.

In a recent bibliometric analysis covering 399 research articles to investigate the evolution of the IP research, Stone-Sabali et al. (2023) identified five clusters of research interest. These focus on (1) the development of the IP construct and possible gender effects, (2) the IP’s relation to medical students, self-esteem, and racial identity, (3) career and organizational psychology, (4) STEM fields, engineering, and sense of belonging, and (5) the IP’s diagnosis and negative effects on individuals (Stone-Sabali et al., 2023).

Some of these clusters implicitly address societal (racial identity), interpersonal (sense of belonging), and institutional (organizational psychology) aspects. Nevertheless, overall, these perspectives seem to be rather isolated from each other and limited in scope as only a small number of contributions suggest that socio-structural and cultural factors may affect the IP (e.g., Bernard et al., 2017; Stone et al., 2018). In psychological research, it is commonly assumed that the IP is largely influenced by an individual’s mindset (e.g., Zanchetta et al., 2020) while influences of family socialization on the development of the IP are moderated by personality traits (e.g., Rohrmann et al., 2020).

From a sociological point of view, it is, on the one hand, reasonable to assume that socialization may partly mediate the relationship between socioeconomic status (SES) and the IP (Sonnak and Towell, 2001, p. 872). On the other hand, an individual’s mindset has to be seen as a product of environmental influences where group-based characteristics might generally have a similarly mediating effect (Nadal et al., 2021). Ibrahim et al. (2023) highlight, e.g., educational differences between mothers and children as potential indicators for the development of the IP. However, to date, little evidence exists in this regard; while some studies have found negative correlations between the IP and SES (e.g., Chrisman et al., 1995; Brauer and Wolf, 2016), questions concerning the role of the IP’s socio-structural correlates remain largely unexplored (Nadal et al., 2021).

Thus, even though Clance et al. (1995) proposed that the IP might be shaped by “interpersonal and social contexts” (80) almost 30 years ago, it seems that the IP is still predominantly understood as a troubling, individual constrain that can be overcome through self-help strategies or therapy (Feenstra et al., 2020). This is underlined by the fact that most research articles stay limited to the examination of personality-based factors, whereas socio-structural characteristics are often disregarded (Feenstra et al., 2020; Anderson-Zorn, 2021).

Looking at the existing empirical evidence base, some critical shortcomings can be identified. Firstly, the existing IP studies are characterized by small, and often highly selective, samples that are almost exclusively utilized in cross-sectional research designs (i.e., measuring only one point in time; Bravata et al., 2019; Mak et al., 2019). This is problematic since, strictly speaking, such research designs do not permit answering questions regarding the general dissemination of the IP or examining variations in its intensity during transitions or other changes in the life course.

Secondly, existing empirical studies on the IP are characterized by large gaps when it comes to reporting essential demographic characteristics, e.g., age or ethnic background (Mak et al., 2019; also see the review by Bravata et al., 2019). On the one hand, this impedes research on the social embeddedness of the IP, i.e., how social structures and status may shape the IP. On the other hand, this contributes to contradictory findings as the results can seldom be compared, while possibilities to link the findings of different studies are likewise limited.

Thus, central questions regarding the role of basic socio-demographic factors in experiencing the IP remain unresolved. This is the case, for example, regarding potential age effects. On the one hand, there is some evidence that suggests that age may be a predictor of the IP (e.g., Ibrahim et al., 2022a,b). However, in their review of the “prevalence” of the IP, Bravata et al. (2019) state that numerous studies have not reported on this very basic demographic feature, while half of those that do report on age found that the IP declines with age while the other half found age not to affect the IP at all. Hence, even the role of age as a very basic socio-demographic characteristic remains “a key open question that future studies evaluating employed populations (rather than just evaluating students)” should address (Bravata et al., 2019).

A third, major shortcoming in empirical IP research is the lack of conceptual clarity and dimensionality. Since the IP was originally conceptualized as a multidimensional phenomenon by Clance and Imes (1978), initial instruments to measure it were designed accordingly. However, this proposed multidimensional factor structure was not reproducible using most IP instruments (Mak et al., 2019). In fact, it is usually the overall sum scores that are calculated, rather than the subscale scores that are reflective of the multidimensional structure of most IP measures (Mak et al., 2019).

Moreover, most IP studies are not very informative in this regard, not necessarily due to a poor questionnaire design or performance, but rather to an absence of appropriate reporting on essential psychometric data (Mak et al., 2019). This can have far-reaching consequences since it may contribute to a situation in which different studies might actually measure different things, contributing to inconsistencies in the empirical database on the IP.

Fourthly, there is a shortage of longitudinal evidence on the IP. While there are studies that cover more than one measurement point (e.g., September et al., 2001; Bouffard et al., 2011; Houseknecht et al., 2019), no profound analyses of the development of the IP over the life course exist; this is the case since the existing longitudinal studies focus on a certain point in the life course (student samples) and cover a maximum of three waves of data collection.

From a perspective interested in understanding the role of social contexts in experiencing the IP, transitions in educational and employment trajectories can be accompanied by recurrent irritations, adjustment challenges, and adaptation periods, making it likely that the IP develops at certain points in the life cycle, although it can decrease in others. Taking this into account, the lack of large-scale longitudinal studies seems to be a significant gap that is particularly striking in light of the voids regarding the effect of age mentioned above.

Connecting these remarks on the empirical evidence base on the IP to the broader trends in IP research (provided by Stone-Sabali et al., 2023), a socially informed perspective contributes to a better understanding of the role of structurally mediated social characteristics in the development and evolution of the IP to advance existing strands in IP research (Feenstra et al., 2020). For a perspective on the IP that includes the role of social context factors contributions that highlight salient socio-cultural factors, such as race and gender, are a fruitful starting point (e.g., Cokley et al., 2015, 2018; Bernard et al., 2017, 2018; Stone et al., 2018; Stone-Sabali et al., 2023).

In summary, it can be concluded that key questions regarding the long-term evolution of the IP as well as its socio-structural correlates remain open. Given the lack of large-scale empirical studies, this is problematic insofar as fairly generalized conclusions regarding the far-reaching psychological implications of the IP seem to be postulated based on a rather limited empirical database.

As mentioned in the introduction, this tendency has proven to be highly influential both within and beyond the academic sphere, creating much noise while providing little tangible evidence. To gain a better understanding of how the shortcomings in IP research regarding its socio-structural correlates are connected to existing IP instruments, the following section provides a brief overview of IP measures.

## 3 Instruments for measuring the IP

There are various instruments available for studying the IP among different target groups. This section provides a summary of the current state of the art in measuring the IP to underscore the need for methodological advancements.

At the international level, four instruments are well-established and widely used, namely the Harvey Impostor Scale (HIPS; Harvey, 1981), the Clance Impostor Phenomenon Scale (CIPS; Clance, 1985), the Perceived Fraudulence Scale (PFS; Kolligian and Sternberg, 1991) and the Leary Impostorism Scale (LIS; Leary et al., 2000).

The HIPS contains 14 items; usually, an overall total score is calculated with this scale rather than the subscale scores that would reflect the multidimensional definition of the measure as postulated by Harvey (1981). Furthermore, the HIPS reveals psychometric weaknesses (i.e., unacceptable low internal consistencies) and insufficient validity (Hellman and Caselman, 2004). Consequently, further use of this scale is not recommended (Brauer and Wolf, 2016).

In contrast, the 20-item CIPS (Clance, 1985) shows very good to excellent psychometric properties in various studies (e.g., Holmes et al., 1993; Simon and Choi, 2018; Ibrahim et al., 2021) and can be regarded as the most widely used instrument. It is based on three theoretical dimensions and assesses self-doubts about one’s own intelligence and abilities (Fake), a tendency to attribute success to chance/luck (Luck), and the inability to admit a good performance (Discount). CIPS has been translated into various languages, resulting in several versions of the scale, most of which have been validated, e.g., in German (Brauer and Wolf, 2016) or Hebrew (Yaffe, 2020). While the CIPS was assumed to be three-dimensional and most studies use the total score (Mak et al., 2019) recent findings from two samples suggest that both strategies reflect the CIPS’ measurement model, since a bifactor model explains the existence of specific subscale factors and a general factor (Brauer and Proyer, 2023).

The PFS (Kolligian and Sternberg, 1991) contains 51 items demonstrating good psychometric properties (Mak et al., 2019). The original PFS validation study proposed a two-factor model (with an overall α of 0.94) and subscale reliabilities for Inauthenticity (α = 0.95) and Self-deprecation (α = 0.85) (Kolligian and Sternberg, 1991). However, Chrisman et al. (1995) found the estimated internal reliability for the PFS to be only 0.57 when applying the Spearman-Brown equation to the 51-item PFS to reduce it to a 20-item CIPS equivalent.

Moreover, CIPS and PFS assess almost the same content and also share comparable relationships to external variables, while they correlate positively, supporting convergent validity (Brauer and Wolf, 2016). Thus, CIPS (20 items) and PFS (51 items) can be considered equivalent in empirical terms, although, for economic reasons, CIPS is clearly favorable.

The LIS (Leary et al., 2000) is the most recent among the internationally established instruments and includes seven items (with a reported α of 0.87). The correlation of the LIS with the other existing instruments (HIPS, PFS, and CIPS) ranges from 0.70 to 0.80, indicating strong evidence for construct validity (Leary et al., 2000). LIS was the first instrument designed as a unidimensional measure, meaning that only for LIS is it logical to calculate an overall score to assess the IP; a practice that researchers, nonetheless, largely apply using all scales described so far (Mak et al., 2019).

In addition to these four internationally established instruments, there are two IP scales of particular relevance since they have been developed for use among children and adolescents; these are the Young Impostor Scale (YIS, Villwock et al., 2016) and the questionnaire du sentiment d’imposture pour enfants et adolescents (QSIEA - Impostor Feelings Questionnaire for Children and Adolescents, Bouffard et al., 2011).

The QSIEA is of particular interest for three reasons: (a) it is specifically designed for use in children and adolescent samples, (b) it has been successfully applied and validated in a longitudinal study (two measuring points), and (c) it is relatively short as it consists of only eight items. QSIEA measures the feelings of threat characterizing the IP without confusing them with associated correlates such as negative perfectionism or perceptions of competence (Chayer and Bouffard, 2010).

QSIEA validation studies showed high internal consistency (α = 0.84, 0.83, respectively) and good temporal stability over a 6-week period (rtt = 0.79), while a confirmatory factor analysis (CFA) showed that all fit indexes unequivocally support its validity (Chayer and Bouffard, 2010; Bouffard et al., 2011). The results for three different samples can be traced back to a single factor that accounts for 57.6% of the variance in IP intensity among adolescents aged 10 to 17 years (Bouffard et al., 2011).

The YIS (Villwock et al., 2016) also contains eight items but uses a quiz-style mode, dichotomously assessing the presence or absence of the IP. In a pilot study with medical students, it was used to describe levels of burnout and the IP and to determine demographic differences in the experiences of these characteristics. Responding “Yes” to five or more of the quiz-style questions was considered a positive finding of the IP.

With the recent explosion of interest in the examination of the IP, it is met with increasing resonance in German-speaking countries, contributing to a growing demand for a stand-alone German-language questionnaire. Consequently, three validated German IP questionnaires exist in the form of the German-language CIPS (GCIPS - German translation of CIPS, Brauer and Wolf, 2016), the Impostor Self-Concept Questionnaire (ISCQ, ISF, Rohrmann et al., 2020) and the Impostor Profile (IPP31, Ibrahim et al., 2022a). These scales are applied from the age of 16 years onwards and serve in the fields of education, work, and organizational development as well as in clinical contexts.

The GCIPS (Clance, 1988) has frequently been used for almost 30 years, e.g., for the study of IP effects on leadership styles (Bechtoldt, 2015), career development (Neureiter and Traut-Mattausch, 2016), or stress and working styles (Rohrmann et al., 2016), without being fully validated and tested concerning its psychometric characteristics. In 2016, Brauer and Wolf (2016) provided a validation study of the GCIPS, applying exploratory factor analysis (EFA) and CFA that yielded three factors (Fake, Luck, and Discount) accounting for 44% of the variance in the IP.

The ISCQ (ISF, Rohrmann et al., 2020) was developed for German-speaking samples and contains 15 items. The ISCQ shows excellent internal consistency (α = 0.93–0.94) and test–retest reliability across 4 weeks (rtt = 0.77). The ISCQ items comprise deceiving others about one’s abilities, external attributions of success, rejections of recognition, and fears of being exposed as an impostor. A total ISCQ score is calculated to assess the IP employing a unidimensional trait. While the ISCQ shows highly positive correlations to other established IP scales (namely CIPS, PFS, and LIS), it is regarded as a “very economical procedure that can be processed or evaluated in about five minutes each” (Rohrmann et al., 2020: 25).

This is not the case for the IPP31 (Ibrahim et al., 2022a) which contains 31 items and is, thus, one of the most comprehensive IP instruments. Being the most contemporary validated German IP instruments, the IPP31 consists of six subscales (Competence Doubt, Working Style, Alienation, Other-Self Divergence, Frugality, and Need for Sympathy) and shows satisfactory internal consistencies of between 0.69 and 0.92, as well as positive correlations with convergent (Neuroticism) and discriminant (Self-Esteem) measures (Ibrahim et al., 2021, 2022a). In addition, an adapted version called IPP30 is also available, which has been validated in English and German and varies only with respect to the change of subscale Frugality to Ambition (Ibrahim et al., 2022b).

Reflecting on the theoretical and methodological overviews it is striking that IP instruments for different age groups differ from each other (e.g., due to item complexity), even though the IP seems to be relevant among the youth, adolescents, and adults alike. Thus, it is not yet possible to study the development of the IP within affected individuals over time in longitudinal (panel) studies starting, for example, in adolescence. Another relevant point connected to this is that most scales are designed for specific social contexts (e.g., educational (QSIEA, YIS) or work environments (CIPS)).

Moreover, attempts to standardize IP assessments have typically included only small numbers of ethnic minorities, which then raises questions about whether current IP instruments are reliably valid for ethnic minority populations (Stone et al., 2018).

In light of this and the open questions mentioned in section 2, large-scale longitudinal studies are of particular relevance for obtaining a more nuanced understanding of the IP. Due to their selective designs and exhaustiveness, the existing IP scales barely fit into most non-psychological surveys that typically include instruments for a wide range of concepts and target diverse study populations. Therefore, the next chapter presents a novel short scale consisting of only three items that can be used among both adolescents and adults in various contexts.

## 4 IPSS-3: a universally applicable IP instrument

This section introduces the Impostor Phenomenon Short Scale (IPSS-3) developed to address the lack of a universally applicable IP instrument. The IPSS-3 is based on the established Impostor Self-Concept Questionnaire (ISCQ, ISF, Rohrmann et al., 2020) mentioned in section 3. A preliminary short version of the original ISCQ called ISCQ-5 (ISF-K; Leonhardt et al., 2025) was chosen as the base instrument for developing the IPSS-3 since it is comparably short and recently developed. Furthermore, it is designed based on a scale with a unidimensional factor structure and shows a highly positive correlation with another established IP scale (CIPS).

In the first step, the ISCQ-5 items were reviewed and linguistically modified to reduce the respondent burden based on recommendations for the question-wording of items used in large-scale longitudinal studies (Lenzner and Menold, 2019). Subsequently, two distinct studies were conducted to (1) examine possibilities for further reducing the number of items of the preliminary ISCQ-5 and (2) to evaluate differences in the measurement quality of the scale version that compromises the simplified and shortened items. A third study was conducted applying the IPSS-3 as a novel 3-item IP measure to gain insights into the socio-structural correlates of the IP, highlighting its potential applications in broader research approaches that include social context variables.

## 5 Materials and methods

### 5.1 Study 1: developing the IPSS-3

To develop the IPSS-3 based on the ISCQ-5, the simplified item formulations were utilized in Study 1 along with the original ISCQ-5 items.

#### 5.1.1 Participants

The sample for Study 1 consisted of *n* = 271 respondents who were recruited in November 2022 through online mailing lists of a major German university and other academic mailing lists, covering different disciplines and universities. A sample size of 250 participants or above was aimed to ensure robust findings and adequate variability for investigating potential differences, contributing to a meaningful progress of the development process. Respondents were predominantly female (80.81%), working (52.40%) or in training/enrolled as students (41.33%), born in Germany (90.41%), and had an average age of 31.89 years (age range of 18 to 71 years).

#### 5.1.2 Instruments

The questionnaire covered items related to employment status, gender, age, educational background, and IP sentiments. The key items applied in this questionnaire were the preliminary shortened ISCQ-5 (original items) and the linguistically simplified formulations that were constructed as the base for determining the items for the IPSS-3. While the reformulated items were placed at the very beginning of the questionnaire, the original ones appeared at the very end.

#### 5.1.3 Procedures

The items were applied in an online questionnaire administered through the survey platform Qualtrics (2023). Before its application, the scale was approved by Goethe University’s Social Sciences Ethics Committee for utilization in both an adult as well as an adolescent sample. The data of Study 1 was used to (1) validate the internal consistency of the ISCQ-5 with initial primary data and confirm its factor structure using CFA, which was expected to be unidimensional similar to the original ISCQ. Furthermore, the data was used to (2) examine the potential effects of the reformulated items on the quality of the scale, and (3) reduce the number of items while maintaining high quality in terms of reliability. The Kaiser-Meyer-Olkin measure (KMO) was used to check whether the data were suitable for factor analysis. The Eigenvalue was used to test whether the first factor contributes the highest variance. To reduce the number of items, their factor loadings were examined to clarify which variables correlate highest with the factor, while only the most important variables were selected.

#### 5.1.4 Results

In Study 1 both IP scales (the one with the original and the one with the reformulated items) showed high loadings on a single factor, as the CFA has revealed, excellent reliability coefficients (see Table 1), and a high correlation (0.97). This indicates that the linguistic item reformulations did not harm the quality of the scale. For the CFA, the maximum likelihood (ML) method was used, whose results were tested for robustness using the maximum likelihood robust (MLR) estimations. The MLR confirmed a single-factor structure for the IPSS-3.

**Table 1:** IPSS-3: Factor loadings (pattern matrix) and unique variances, ML estimator.

Variables	Factor 1	Uniqueness	Kaiser–Meyer–Olkin values
Afraid of failing again at every request	0.87	0.25	0.68
Fear that others notice lack of knowledge/skill	0.82	0.33	0.75
Fear of not meeting the expectations of others	0.85	0.27	0.69
Eigenvalue	2.15		
Variance	0.72		
Total			0.70
Factor rotation matrix: Factor 1 = 1.00			
MLR: Retained factors = 1			

To shorten the instrument further for use in large-scale and longitudinal studies, the reliability was determined for various reduced combinations of the reformulated items. While two combinations showed slightly higher alpha values (0.83 for ISCQ-5 items 1, 2, 3, and 1, 3, 5), all others exhibited quite similar alpha values ranging from 0.79 to 0.82. Given the rather marginal differences between these alternatives, and especially because of the almost identical wording of some items (that, of course, increases reliability), the selection was not based solely on the statistical indicators. Instead, preference was given to those items that, in combination with each other, speak consistently to the core elements of the IP:

(1) being afraid of failing at every request,
(2) fears others notice a lack of knowledge/skill.
(3) feelings of social inadequacy associated with sentiments of not belonging/meeting others’ expectations.

The IPSS-3 compromises these three (modified) items to represent a novel time-efficient IP instrument that is shown in Table 2 along the recommended response categories. Table 1 provides information concerning the factor loadings and unique variances of the IPSS-3, showing high reliability (α = 0.80, Ω = 0.80), that is only marginally below that of the preceding ISCQ-5 (original version: α = 0.88, Ω = 0.88). Given the fact that the IPSS-3 is based on merely three items (instead of five), the declined reliability can be regarded as acceptable (Rammstedt and Beierlein, 2014).

**Table 2:** IPSS-3: Items and response categories.

1	Though I am often successful, I become afraid of failing again at every request.
2	Sometimes I fear others will notice how much knowledge and how many skills I actually lack.
3	I often fear not being able to meet the expectations of others, even though I have already achieved a lot.
Response categories	
(1) does not apply at all	
(2) mostly does not apply	
(3) does not really apply	
(4) applies a bit	
(5) mostly applies	
(6) absolutely applies	

The factor structure is confirmed by robust results regarding the root mean squared error of approximation (RMSEA = 0.00), comparative fit index (CFI = 1.00), and Tucker-Lewis index (TLI = 1.00) based on a CFA using a structural equation model (SEM), whose results are replicated for all samples of studies 1 to 3 (see Supplementary Table A5).

In summary, the results of Study 1 indicate that the IPSS-3 exhibits a high validity, supported by its similarity to the ISCQ-5 in terms of factor loadings and reliability scores. Furthermore, the IPSS-3 is based on the ISCQ as a validated 15-item IP measure that demonstrates strong correlations with multiple other established IP instruments.

### 5.2 Study 2: validating the IPSS-3

In Study 2, a second sample was used to confirm the measurement quality of the IPSS-3, assess its construct validity, and evaluate the scale in terms of its practicality, comprehensibility, and acceptability drawing on a second set of primary data collected among German adolescents. This sample selection was chosen to ensure that the IPSS-3 is suitable for a younger target group than those at the core of current IP research: While existing IP instruments predominantly refer to students or employees, the sample of study 2 utilizes an age group in which particular sharp differences may exist, e.g., due to attending different types of school before continuing with higher education.

#### 5.2.1 Participants

The sample of Study 2 consisted of *n* = 427 adolescents living in Germany who were recruited by an online panel provider in February 2023. The respondents had an average age of 17.99 years (age range of 16 to 19 years), were predominantly female (73.30%), and had no migrant origin (94.84% were born in Germany, while for 91.16%, both parents were born in Germany).

The sample was drawn to test the suitability of the IPSS-3 for different educational levels and, therefore, it consisted of students from different school types: 7.96% from lower secondary/comprehensive/upper secondary school, 49.65% from higher secondary school, and 22.48% from vocational schools.

#### 5.2.2 Instruments

The questionnaire covered a large range of items focusing on the educational trajectories of the respondents, attitudes toward political topics and political participation, the IPSS-3, the ISCQ-5 (original version), and two scales capturing constructs that are commonly considered to correlate with the IP, namely (1) External Locus of Control (e.g., Clance and Imes, 1978: 242; Byrnes and Lester, 1995; Rohrmann et al., 2016) and (2) Self-Esteem (e.g., Oriel et al., 2004; Neureiter and Traut-Mattausch, 2016; Cokley et al., 2018; Naser et al., 2022).

For External Locus of Control, the two items for assessing the external control beliefs of the Internal-External Control Beliefs Short Scale (IE-4; Kovaleva, 2012) were utilized. Here, a convergent (positive) correlation with the IPSS-3 was expected since individuals with the IP attribute control externally. The Brief Rosenberg Self-Esteem Scale (BRS-5; Monteiro et al., 2022) was applied as a second validation scale. The BRS-5 comprises five items that were expected to discriminantly (negatively) correlate with the IPSS-3. Furthermore, the ISCQ-5, with its original item formulations, was included in the questionnaire to confirm the quality of the IPSS-3 in terms of convergent validity by comparing the results of both scales, based on the fact that the ISCQ-5 correlates strongly with the ISCQ-15 (0.85).

#### 5.2.3 Procedures

The questionnaire was administered online through the survey platform Qualtrics (2023). The online panel provider Bilendi and respondi distributed the link to participants among a selected sample of adolescents who answered the survey via smartphone, tablet, laptop, or desktop computer. The IPSS-3 appeared in the first quarter of the questionnaire along with instruments for the respondents’ self-evaluations (e.g., self-efficacy). The validation scales and the ISCQ-5 were implemented at the very end of the questionnaire.

#### 5.2.4 Results

In Study 2, the IPSS-3 showed high loadings on a single factor, while the reliability revealed lower but still acceptable values (α = 0.76; Ω = 0.77). Table 3 lists the correlations of the IPSS-3 with the three validation scales, ISCQ-5, IE-4 external, and BRS-5. The correlations were found to be significant and in the expected direction, suggesting that there is a definite and reasonable association.

**Table 3:** IPSS-3: Correlations of validation scales.

	IPSS-3	
ISCQ-5Impostor Self-Concept Questionnaire (preliminary short version)	0.88	^***^
IE-4 ExternalInternal-External Control Beliefs Short Scale	0.30	^***^
BRS-5Brief Rosenberg Self-Esteem Scale	−0.47	^***^

The high correlation between the reformulated and reduced items of the IPSS-3 and the original ISCQ-5 underlines the high similarity of both scales, supporting the validity of the IPSS-3. The lower correlation with the other two concepts emphasizes that they are equally associated with each other but still separate constructs (Ratner, 2009).

In summary, the findings of Study 2 underline that the IPSS-3 does not merely measure what it is supposed to but does so in a highly efficient manner. Thus, it represents the first validated IP short scale. Moreover, meaningful deviations of IP values among different school tracks (lower scores in lower tracks, higher scores in higher tracks, especially in those including transitions, i.e., upper secondary and vocational schools) indicate that the IPSS-3 can be used to capture IP sentiments in adolescence. Furthermore, the overall reasonable distribution of IP scores across all school types shows that the IPSS-3 can be considered a universal instrument that can be used regardless of the respondents’ educational backgrounds (for lower secondary/comprehensive/upper secondary schools: mean = 11.89 (SD = 3.48), median = 12, skewness = −0.10, kurtosis = 2.42; for higher secondary: mean = 12.25 (SD = 3.66), median = 13; skewness = −0.46, kurtosis = 2.69; for vocational schools: mean = 12.16 (SD = 3.31); median = 12, skewness = −0.14, kurtosis = 2.53).

### 5.3 Study 3: understanding IP’s socio-structural correlates

Study 3 was conducted to confirm the IPSS-3’s effective performance in and general applicability to large-scale surveys encompassing diverse study populations. Furthermore, the study sought to acquire a deeper understanding of the socio-structural factors associated with the IP, utilizing a distinct primary data set. The respondents were recruited in September 2023 by the online panel provider Bilendi and respondi.

#### 5.3.1 Participants

Two German adult populations were utilized: (1) a general sample, and (2) a distinct Turkish oversample. The total sample consisted of *n* = 865 adult respondents, with or without migrant origin (general sample: *n* = 599; Turkish oversample: *n* = 266). Respondents were nearly evenly distributed in terms of gender (55.26% male), with the majority being either (self-)employed (60.46%) or retired (13.76%).

They had an average age of 43.44 years (age range of 19 to 88 years), while 48.1% were of migrant origin meaning that either they themselves or at least one parent or grandparent was not born in Germany; although this was, by definition, the case for all respondents of the Turkish oversample, in the general sample, the share (25.0%) was almost identical to that of the general German population (24.3%; Destatis, 2023).

#### 5.3.2 Instruments

The questionnaires included a series of instruments concerning social networks, attitudes toward political topics, political self-positioning and participation, and items on emotional and social well-being, including the IPSS-3. In addition, comprehensive information on the socio-demographic characteristics of the respondents was collected. To reduce respondent burden and to unify the response categories throughout the questionnaire, the IPSS-3 was administered with only five answer options (instead of the original six). These options ranged from 1, indicating “does not apply at all,” to 5, indicating “completely applies.” As a result, the IP scores in this study ranged from a minimum of 3 to a maximum of 15.

#### 5.3.3 Procedures

The IPSS-3 was implemented in the second of ten question blocks of the survey after some basic socio-demographics were collected. In the first step, the data of both the general as well as Turkish oversamples were used separately to assess the key performance indicators of the IPSS-3 regarding the duration, reliability, and factor loadings. This was used to confirm the results of Study 2 in both samples while highlighting the time efficiency of the IPSS: median duration 17–19 s, mean < 20 s (see Supplementary Table A1 in the Appendix). In the following step, the data of both sub-samples were merged into a single data set that was used for the following analysis.

#### 5.3.4 Results

The data from Study 3 was utilized to examine the relevance of socio-structural characteristics on the IP. To this end, the variables were recoded to allow the comparisons of IP intensity by gender (male vs. non-male), migration origin (yes vs. no), and educational attainment (academic vs. non-academic).

Table 4 provides a concise summary of the central findings for each of the most significant comparisons (gender: binary; migration: yes vs. no; education: non- vs. academic) based on t-test comparisons for significant differences in IP mean values. The findings suggest that there are significant differences in the intensity of the IP based on gender (whereby the group “Female/Diverse” shows higher values; mean difference: −0.66, *p* = 0.01) and between individuals with and without a migrant origin (whereby the group possessing a migration origin shows higher values; mean difference: −0.80, *p* = 0.01). However, there were no significant differences in IP mean values found between individuals with and without a university degree (mean difference: −0.26, *p* = 0.26).

**Table 4:** IPSS-3: Group comparisons, mean values, and t-tests.

Impostor Phenomenon	All	Gender		Migration origin		Education (university degree)	
		Male	Female/diverse	No	Yes	No	Yes
Mean	7.92	7.62	8.28	7.53	8.33	7.84	8.10
sd	3.12	3.02	3.19	3.12	3.06	3.12	3.09
Diff.			−0.66		−0.80		−0.26
se			0.21		0.21		0.23
*p*-value			< 0.01		< 0.01		0.26
Hedges’s *g*			−0.20		−0.25		−0.08
[95% Conf. Int.]			−0.34–0.07		−0.39–0.13		−0.23 0.06
*N*	865	478	387	449	416	592	267

The findings are similar when the variables are modified, i.e., when gender is measured using only males vs. females (omitting diverse), migration origin is divided by generational status (none, first, second, third generation), and education is differentiated into three (secondary school, high school, university graduation) or four (adding no education) groups. The results for these additional calculations, as well as boxplots illustrating the distribution of IP values for these groups, are listed in the Appendix (Supplementary Figures A1–A3). Overall, these results indicate an unequal distribution of IP intensity among different social groups based on gender and migrant origin, while education, as such, does not seem to correlate with the IP.

## 6 Discussion, limitations, and future research

This paper presents the results of three separate studies: Study 1 outlined the development of the IPSS-3 as a novel three-item short scale for measuring the IP, Study 2 focused on its validation, and Study 3 underscored the overall significance of a perspective interested in the role of social contexts in fostering a more nuanced understanding of the IP.

The IPSS-3 shows an excellent performance in terms of construct validity as it is based on the validated 15-item ISCQ (Rohrmann et al., 2020). It represents a significant advancement in the current IP assessment instruments as it can facilitate large-scale research designs exploring factors such as the overall occurrence, socio-structural correlates, and long-term evolution of the IP, thus highlighting the importance of societal, institutional, and interpersonal contexts.

The excellent quality criteria of the IPSS-3 in terms of its high reliability and factor loadings indicate that it is ready for implementation in future data collections as revealed in Study 1. This also applies to the results concerning the validity of the IPSS-3 gained in Study 2. Here, definite relationships between the IP and two external criteria were used to assess the accuracy of the IPSS-3: Firstly, an instrument for External Locus of Control, and secondly one for Self-Esteem. These evaluations underline that the IPSS-3 measures what it is supposed to measure in an unprecedented, time-efficient manner.

The results related to the socio-structural correlates of the IP highlighted by the findings of Study 3 point to the relevance of socio-demographic characteristics in its experience. These results show a significant difference in the IP by gender that is consistent with numerous other studies (e.g., Lester and Moderski, 1995; Cusack et al., 2013; Hutchins and Rainbolt, 2017).

Nonetheless, taking into account the general evaluation of IP research outlined in section 2, the effect of gender must still be considered one of the key blank spaces in IP research since contradictory findings continue to be reported; the number of studies detecting a gender effect is somewhat equal to the number of studies that do not, making the answer to this question ambiguous (Bravata et al., 2019).

While this may partly be explained by the fact that most IP studies rely on small and highly selective samples, the picture seems to be more complex here. In fact, findings by Fassl et al. (2020) indicate that the IP strongly correlates with the perception of gender attributes rather than gender itself (showing that the IP is positively correlated with negative aspects of femininity and negatively correlated with positive aspects of masculinity). This underlines that further research is needed in this respect that includes not only socio-structural factors but also instruments to capture the perception and evaluation of gender-based characteristics (also see Cokley et al., 2015).

Previous studies also suggest that the IP may be particularly prevalent among individuals belonging to minority groups. Some studies have demonstrated that the IP is common among ethnic minorities or individuals of migrant origin (e.g., Ewing et al., 1996; McClain et al., 2016; Bernard et al., 2018).

In line with this, the results of Study 3 indicate significant differences in the IP mean scores between individuals with versus those without migrant origin, with scores that are, on average, higher for individuals with migrant origin (8.33) compared to those without (7.53).

Given that most of the IP studies that investigate migrant/minority populations are rather small in sample size, while not including large comparison groups, the findings of Study 3 add new comparative evidence to the current state of IP research. Nonetheless, large-scale representative surveys seem to be of high importance to obtain robust data that allows meaningful group comparisons to gain a clearer picture regarding the relationship between migrant origin and the IP.

A similar argument can be made concerning the fact that a large proportion of existing IP studies is based on student samples (Bravata et al., 2019 report a weighted mean age of 20 years), suggesting that academics might be particularly affected by the IP. However, the findings of Study 3 do not indicate that a university educational background, itself, is a factor for high IP intensity. Rather, it might be beneficial to understand the phase of studies as a stage in which the IP could be particularly pronounced, instead of considering the IP as a solid personality trait. This assumption is supported by the findings of Lee et al. (2022), who found that older age groups and people currently not in-training report lower IP scores in a sample of graduate students and professionals in science, technology, engineering, mathematics, and medicine. Furthermore, MacInnis et al. (2019) demonstrate the importance of subjective SES for understanding the IP, underlining that students who view themselves to be relatively lower in SES compared to their peers, reported higher IP scores. In this respect, studies examining the development and long-term evolution of the IP could be promising to gain a better understanding of the role of transitions, social compositions, and institutional contexts, as well as age effects.

As this is clearly beyond the scope of this work, this paper instead offers an impression concerning some central gaps in the existing IP literature, underlining that it is worth examining socio-structural features in more detail. Here, further research seems to be deeply needed, especially given the huge gap between the ongoing pop-cultural addressing of the IP and the limited existing scientific evidence (Feenstra et al., 2020).

That being said, it is worth pointing out that there are also several limitations to the results of this paper. First of all, this paper presents data that is only related to the German version of the IPSS-3 and thus recommends using only this version. Future studies might validate English or other language versions of the scale. Furthermore, all findings reported in this study are based on self-reports, meaning that correlations with real-life criteria or other sources of data (e.g., peer reports; grades) are not provided. As the study populations were recruited through a convenience sample as well as a panel provider, the findings can not be generalized to the general population. Thus evidence on a large scale level is still missing, meaning that larger data collections covering the IPSS-3 represent a particularly tempting field.

Nevertheless, the IPSS-3 represents a fruitful supplement to the methodological instruments for examining the IP, suitable for addressing some central gaps in the empirical database. Therefore, it is essential to have the particular strengths of the IPSS-3 for innovative empirical research in mind: The IPSS-3 is well-suited for large-scale studies when time and resources are limited, e.g., due to highly comprehensive survey designs covering a wide range of topics or survey experiments. On the other hand, the IPSS-3 is not designed for diagnostic purposes or to capture the subdimensions of the IP (e.g., for specific interventions or training).

## 7 Conclusion

In conclusion, the IPSS-3 introduced in this paper represents a validated, time-efficient, and universally applicable instrument for capturing IP sentiments that is ready to be implemented in large-scale assessments of the occurrence of the IP, including longitudinal survey designs. Thereby, the IPSS-3 can facilitate a multitude of research ventures examining the importance of social contextual factors in the development and treatment of the IP to gain a more nuanced understanding of the role of societal, institutional, and interpersonal aspects.

## Data availability statement

The datasets presented in this study can be found in online repositories: Open Science Framework: www.osf.io/vx68t/ - doi: 10.17605/OSF.IO/VX68T.

## Ethics statement

The studies involving humans were approved by Goethe University’s Social Sciences Ethics Committee. The studies were conducted in accordance with the local legislation and institutional requirements. Written informed consent for participation in this study was provided by the participants’ legal guardians/next of kin.

## Author contributions

MJ: Conceptualization, Data curation, Formal analysis, Investigation, Methodology, Validation, Writing – original draft, Writing – review & editing.
