# Correction: Impostor phenomenon short scale (IPSS-3): a novel measure to capture impostor feelings in large-scale and longitudinal surveys

**DOI:** 10.3389/fpsyg.2025.1692257

**Published:** 2025-09-23

**Authors:** Max P. Jansen

**Affiliations:** ^1^Faculty of Social Sciences, Goethe University Frankfurt, Frankfurt am Main, Germany; ^2^Institute for Social Research (IfS), Goethe University Frankfurt, Frankfurt am Main, Germany

**Keywords:** impostor phenomenon, short scale, large-scale surveys, longitudinal research, social identity, social context

Leonhardt et al., 2025 was not cited in the article. The citation has now been inserted in section “4 IPSS-3: a universally applicable IP instrument”, **paragraph 1** and should read:

“A preliminary short version of the original ISCQ called ISCQ-5 (ISF-K; Leonhardt et al., 2025) was chosen as the base instrument for developing the IPSS-3 since it is comparably short and recently developed. Furthermore, it is designed based on a scale with a unidimensional factor structure and shows a highly positive correlation with another established IP scale (CIPS).”

The complete reference can be found below:

Leonhardt, M., Klug, K., Kamsties, N., and Rohrmann, S. (2025). Kurzversion des impostor-selbstkonzept-fragebogens (ISF-K): entwicklung und validierung eines ökonomischen inventars zur erfassung des impostor-phänomens. *Diagnostica*. doi: 10.1026/0012-1924/a000349

The original version of this article has been updated.

