# Supplementary material for: Impostor phenomenon short scale (IPSS-3): a novel measure to capture impostor feelings in large-scale and longitudinal surveys
Source: Front Psychol. 2024 Nov 12;15:1358279. doi: 10.3389/fpsyg.2024.1358279 (PMC11635767; doi:10.3389/fpsyg.2024.1358279)
Supplement: Supplementary file 1 [file Data_Sheet_1.PDF]

Supplementary Material

1 Supplementary Figures and Tables

1.1 Supplementary Tables

Supplementary Table A1 | IPSS-3: Performance indicators for adolescents and adult sample (main, Turkish oversample).

	Study 2 adolescents	Study 3 main sample	Study 3 Turkish oversample
Factor loading	1	1	1
Alpha	0.76	0.86	0.82
Omega	0.77	0.86	0.83
Duration (seconds)			
mean	-/-	25.27	27.48
median	-/-	19.29	17.71

Supplementary Table A2 | IPSS-3: Comparison of IP mean values by gender (male vs. female; excluding diverse), t-test.

Impostor Phenomenon	All	Gender (excluding diverse)	
		male	female
mean	7.92	7.62	8.26
sd	3.12	3.02	3.18
diff.			-0.63
se			0.21
p-value			0.01
N	863	478	385

Supplementary Table A3 | IPSS-3: Pairwise comparison of IP mean values by migrant generation, ANOVA (Bonferroni adjusted).

	Impostor Phenomenon			
	Migrant origin	0<br>none<br>(= ref.)	1<br>first generation	2<br>second generation
0	none (= ref.)			
1	first generation	0.47<br>1.00		
2	second generation	0.78<br>1.00	0.31<br>1.00	
3	third generation	0.81<br>0.01	0.34<br>0.01	0.03<br>1.00

Supplementary Table A4 | IPSS-3: Pairwise comparison of IP mean values by educational degree, ANOVA (Bonferroni adjusted).

Impostor Phenomenon	Educational degree	0 no degree (= ref.)	1 secondary school	2 high school
0	no degree (= ref.)			
1	secondary school	-1.99		
		1.00		
2	high school	-1.62	0.37	
		1.00	0.94	
3	university	-1.57	0.42	0.05
		1.00	0.61	1.00

Supplementary Table A5 | IPSS-3: Measurement Invariance Examination, Confirmatory Factor Analysis based on Structural Equation Model.

(sub)sample population	All	Study 1: all	Study 1: employees	Study 1: students	Study 2: adolescents	Study 3: employees
RMSEA	0.000	0.000	0.000	0.000	0.000	0.000
CFI	1.000	1.000	1.000	1.000	1.000	1.000
TLI	1.000	1.000	1.000	1.000	1.000	1.000
N	916	267	111	139	126	523

1.2 Supplementary Figures

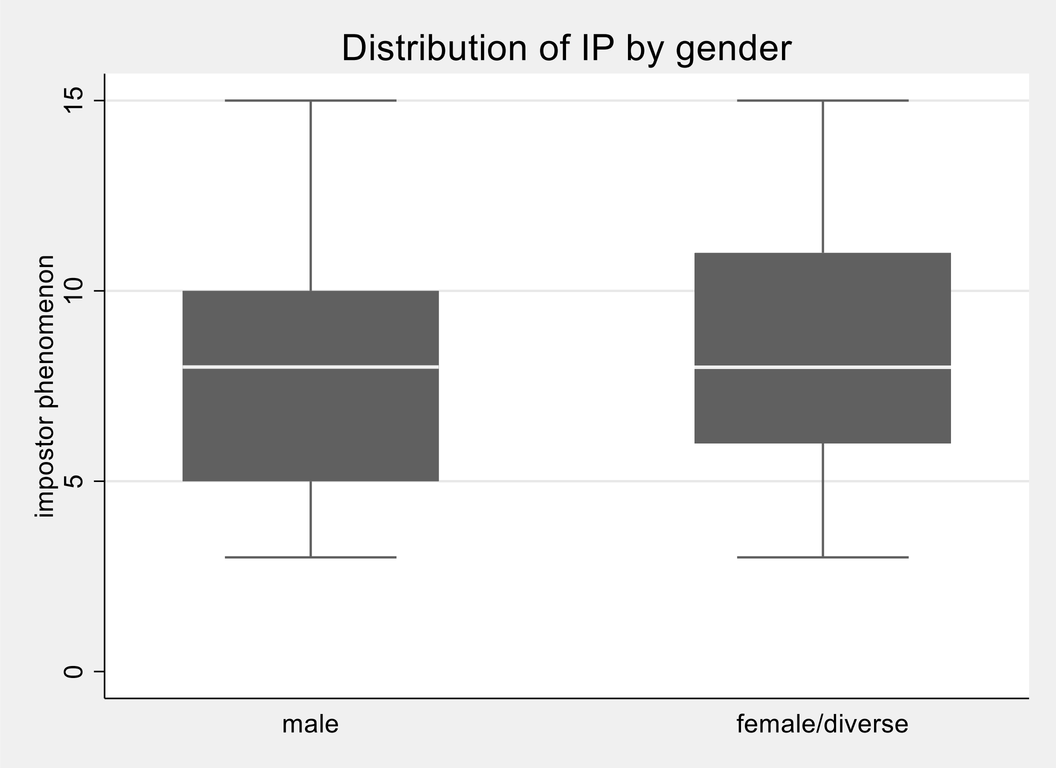

Supplementary Figure A1. IPSS-3: Distribution by gender (male vs. female/diverse).

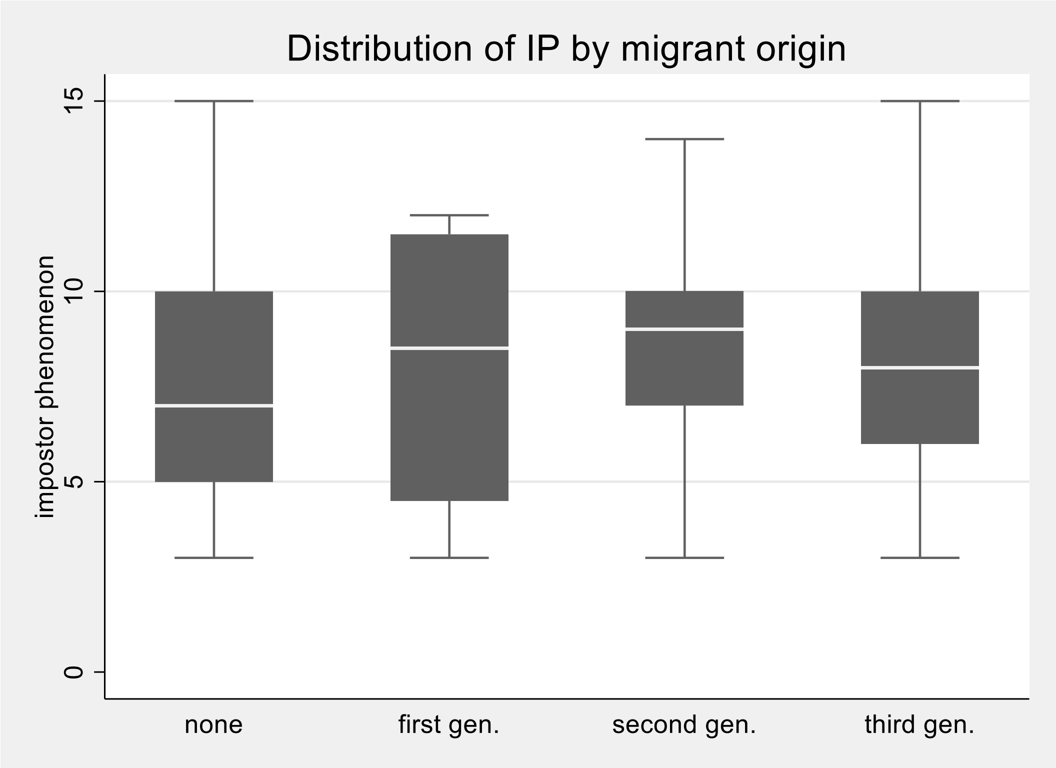

Supplementary Figure A2. IPSS-3: Distribution by migrant origin (generational status).

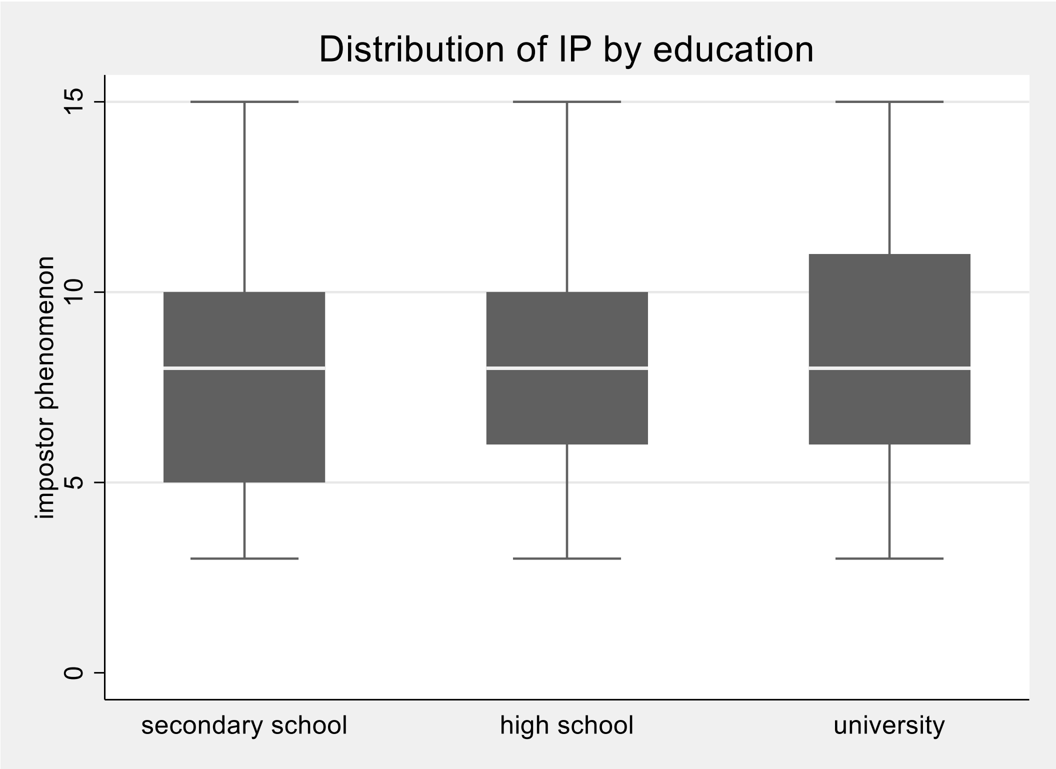

Supplementary Figure A3. IPSS-3: Distribution by educational degree (three groups).
